# Immunotherapy of Tumors with α_2_-Macroglobulin-Antigen Complexes Pre-Formed *In Vivo*

**DOI:** 10.1371/journal.pone.0050365

**Published:** 2012-11-30

**Authors:** Sudesh Pawaria, Laura E. Kropp, Robert J. Binder

**Affiliations:** 1 Department of Rheumatology, University of Pittsburgh, Pittsburgh, Pennsylvania, United States of America; 2 Graduate School of Public Health, University of Pittsburgh, Pittsburgh, Pennsylvania, United States of America; 3 Department of Immunology, University of Pittsburgh, Pittsburgh, Pennsylvania, United States of America; Duke University Medical Center, United States of America

## Abstract

The cell surface receptor CD91/LRP-1 binds to immunogenic heat shock proteins (HSP) and α_2_M ligands to elicit T cell immune responses. In order to generate specific immune responses, the peptides chaperoned by HSPs or α_2_M are cross-presented on MHC molecules to T cells. While the immunogenic HSPs naturally chaperone peptides within cells and can be purified as an intact HSP-peptide complex, the peptides have had to be complexed artificially to α_2_M in previous studies. Here, we show that immunogenic α_2_M-peptide complexes can be isolated from the blood of tumor-bearing mice without further experimental manipulation *in vitro* demonstrating the natural association of tumor antigens with α_2_M. The naturally formed immunogenic α_2_M-peptide complexes are effective in prophylaxis and therapy of cancer in mouse models. We investigate the mechanisms of cross-presentation of associated peptides and co-stimulation by APCs that interact with α_2_M. These data have implications for vaccine design in immunotherapy of cancer and infectious disease.

## Introduction

CD91/LRP-1 is a cell surface receptor in the scavenger receptor family that binds to and internalizes a number of diverse ligands including the heat shock proteins gp96, hsp70, hsp90 and calreticulin and the blood protein α_2_M [1]–[3]. The binding to, and internalization of the HSPs or α_2_M by CD91/LRP-1 leads to processing of peptides that are chaperoned by these proteins and the presentation of the resultant peptides on MHC I and MHC II molecules [1], [2], [4]–[6]. Other receptors for the immunogenic HSPs [7] and α_2_M [8] have been proposed as well as other mechanisms for HSP uptake [9]. A comprehensive discussion on the HSP receptors has been reviewed elsewhere [10]. We and others have used this pathway of cross-presentation to prime T cell responses to peptide antigens expressed by tumors or pathogens. Immunization with CD91/LRP-1 ligands therefore provides the opportunity to vaccinate against, and treat, cancer and infectious disease [11]–[13].

The immunogenic HSPs naturally chaperone peptides within cells and thus the HSP-peptide complex can be purified from cells as an intact immunogenic unit from tumor cells. This approach has two clinically beneficial tenets; i) it is unnecessary to define the antigens expressed by each tumor, and ii) T cells to multiple epitopes are generated [14] which decreases the possibility of tumor escape through loss of antigen expression. Alternatively peptides of choice can be artificially complexed to HSPs *in vitro* which can be used to elicit T cell immune responses with the respective and defined specificities [15]. Similar to the immunogenic HSPs, α_2_M has chaperone properties, sequestering proteins and polypeptides within its basket-like conformation. By doing so it performs various functions in the blood such as inhibition of proteinases and transport of cytokines [16]. However, α_2_M is a secreted protein made primarily by hepatocytes and a smaller proportion by macrophages [16]. Due to differences in localization of α_2_M (blood) and tumor antigens (cell associated), α_2_M-antigen complexes cannot routinely be purified directly from the tumor as is done for HSPs. Thus complexing of singular, defined tumor-derived peptide antigens to α_2_M *in vitro* has been the principle way of eliciting immune responses that target murine tumors [4]–[6].

In this manuscript three areas of investigation are conducted; first, we test whether α_2_M complexed to a bulk preparation of tumor-derived peptides (representing the entire antigenic repertoire of the tumor) can be used in therapy of established tumors in mice, circumventing the identification of tumor specific antigens. Second, we test if α_2_M can bind to tumor antigens released into the tumor microenvironment (and beyond) and whether these pre-formed α_2_M-tumor antigen complexes are immunogenic. Third, we examine the two signals required for T cell priming with respect to the APC-α_2_M interaction; cross-presentation and co-stimulatory cytokines. Our studies conclude that, α_2_M associates with tumor antigens *in vivo* and that these α_2_M-peptide complexes are therapeutically effective against established tumors. α_2_M activates APCs to secrete pro-inflammatory cytokines and introduces peptides into the classical MHC I pathway for antigen processing and presentation. Our studies provide a unique vaccination strategy for the treatment of cancer and provide an alternate view on the long held idea that cellular components of blood are the only entities that can transfer tumor immunity [17].

## Methods

### Cell lines and mice

The RAW264.7 cells were obtained from American Type Culture Collection (ATCC, Manassas, VA) and maintained as recommended. Mice were obtained from Jackson Laboratory (Bar Harbor, ME) and maintained in the DLAR facility at the University of Pittsburgh, Pittsburgh PA. All mice usage was covered under existing and approved IACUC protocols.

### Phosphorylation of CD91/LRP-1

RAW264.7 cells were grown in P6 culture dishes and pulsed with 200 µg/ml mouse α_2_M or 10 ng/ml LPS or PBS for 30 min. The cells were washed with cold PBS and lysed in 1 ml of NP40 Lysis buffer (150 mM NaCl, 50 mM Tris, 5 mM EDTA, 0.5% Nonidet P-40, 5 mM iodoactetamide, 1 mM NaF, 1 mM glycerol 2-phosphate disodium, 1 mM sodium orthovanadate, 2 mM phenylmethylsulfonyl fluoride, 0.5% octylβ-D-glucopyranoside). Lysates were cleared by centrifugation at 10,000 g for 15 min in a microcentrifuge, incubated with 10 µg of mouse anti-CD91/LRP-1 β-chain antibody (11H4, ATCC) for 1 h on ice, and subsequently for 1 h with 100 µl of 10% protein G-Sepharose at 4°C on an agitator. Sepharose beads were collected by centrifugation and washed 4 times with NP40 lysis buffer. Immunoprecipitates were boiled for 3 min in 0.5 M Tris/Cl, pH 6.8, 10% glycerol, 5% β-mercaptoethanol, 2.3% SDS, and 0.025% bromphenol blue (SDS sample buffer) and resolved by SDS-PAGE and transferred to PVDF membranes for immunoblotting. Membranes were blocked for 1 h at room temperature in 10 mM Tris/Cl, pH 7.4, 150 mM NaCl, 0.2% Tween 20 (TBST) containing 5% dried milk and incubated with 4G10/PY20 anti-phosphotyrosine antibodies in TBST 5% milk for 1 h at room temperature. Blots were washed twice for 10 min with TBST and then incubated for 1 h with horseradish-peroxidase-conjugated polyclonal IgG antibody (Sigma, St. Louis, MO) in TBST and washed as before. Reactive proteins were visualized by ECL (Amersham Pharmacia Biotech, Piscataway, NJ). The protein size was confirmed by molecular weight standards (Bio-Rad, Hercules, CA).

### Cytokine array

Splenic DCs were obtained from C57BL/6 mice by CD11c^+^ selection using Miltenyi pan DC kit (Miltenyi Biotec Inc., Auburn CA). DCs were incubated for 20 h with 200 µg/ml mouse α_2_M or PBS. Supernatants were harvested and assayed by the Proteome Profiler Array (Mouse Cytokine Array Panel A, R&D systems Inc., Minneapolis, MN) according to the manufacturer's instructions. Each membrane consists of selected capture antibodies that are spotted in duplicate on nitrocellulose membranes. A total of 40 cytokines are analyzed on each membrane. TNF-α ELISA (eBiosciences, San Diego, CA) was performed, in the presence or absence of cardamonin. Cardamonin was obtained from TOCRIS Biosciences (Ellisville, MS) and used at a concentration of 10 µM. Endotoxin was undetectable in all α_2_M preparations as tested by the LAL assay and LPS-pulsed DCs provided a different and, for several cytokines such as IL-1β, a mutually exclusive cytokine profile [18].

### Cross-presentation assay

Cross-presentation assays were performed as previously described [4]. B3Z is a T cell hybridoma that recognizes SIINFEKL and was provided by Nilabh Shastri (University of California, Berkeley, CA) [19]. B3Z has the β-gal gene under the IL-2 promotor and recognition of SIINFEKL by B3Z leads to the production of β-gal. Briefly, 20,000 bone marrow-derived dendritic cells were incubated with protein, peptide, or protein-peptide complexes with B3Z cells for 20 h. Incubations were performed in 200 µl in 96-well plates. Cells were harvested, washed, permeabilized and incubated with chromogenic β-galactosidase (β-gal) substrate chlorophenol red-β-D-galactopyranoside (SIGMA, St Louis MO). Absorbance at 595 nm was measured. Peptides were synthesized by Genemed Synthesis Inc., (San Antonio, TX). The peptide complexed to α_2_M was ova19 (SGLEQLESIINFEKLTEWTS). Direct or cell surface presentation is not possible until the peptide is processed intracellularly. Gp96 was purified from mouse tissue [4] and α_2_M from serum under GLP conditions [20]. Purified α_2_M was analyzed by SDS-PAGE and was deemed to be apparently homogenous. The identity of α_2_M was confirmed by immunoblotting with an α_2_M-specific antibody which does not cross-react with murinoglobulin (SIGMA, St. Louis, MO). Peptides were complexed to the chaperone as previously described [15], [20]. By using mild thermal manipulation to make the α_2_M-peptide complexes, approximately 1% of the α_2_M is loaded with peptide [data not shown, 15]. Cross-presentation assays were performed in the presence of the proteasome inhibitor lactacystin or brefeldin A (BFA), an inhibitor of vesicular traffic. Lactacystin (SIGMA) was used at 100 µM for 2 h prior to addition of proteins or peptides and at 10 µM for the remainder of the time of the incubation. Cells were treated with BFA (SIGMA) at 6.0 µg/ml for 3 h prior to incubation with proteins and at 0.6 µg/ml for up to 12 h. Maintenance of the lactacystin or BFA block did not affect B3Z function.

### Tumor rejection assays

In the tumor prophylaxis assays, BALB/c mice were immunized with 20 µg of α_2_M subcutaneously. One week later, mice were challenged intradermally with 100,000 MethA or CMS5 tumor cells. In this procedure, either tumor cell type was harvested from culture and washed three times with PBS. Cell viability was determined to be >95% and diluted to 100,000 cells per 100 µl. Mice were challenged with tumor cells. Tumor growth was measured on two axes. In the tumor therapy experiments, mice were challenged with 2×10^6^ CMS5 cells as described above. Tumors were allowed to progress to 3×3 mm in diameter (day3–4) at which point they were treated with α_2_M or gp96 with or without peptide or peptides alone as indicated. Treatments consisted of 20 µg protein/complex every other day for a total of 4 treatments. Tumor growth was measured on two axes.

### Statistical analysis

P values were obtained by two tailed paired t-test. Error bars in graphs represents standard deviation from the mean in duplicates.

## Results

### Therapeutic efficacy of α_2_M complexed to tumor-derived peptides *in vitro*

**Figure 1:**
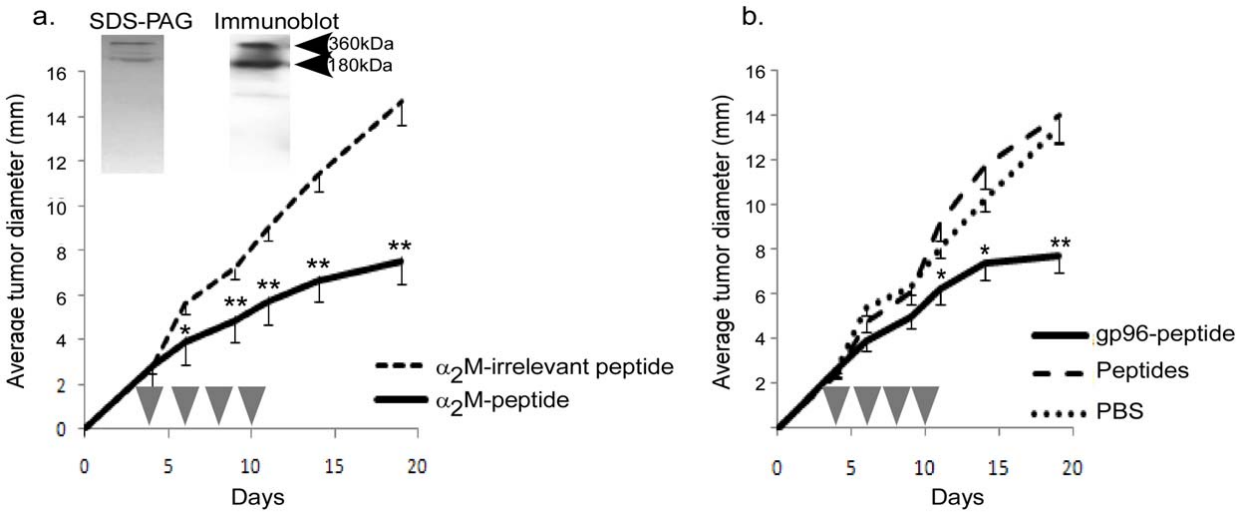
α_2_M-tumor peptides complexes are effective in immunotherapy. a&b) cohorts of BALB/c mice were challenged with the CMS5 tumor as described in Methods. Mice bearing 3×3 mm diameter tumors were treated with α_2_M complexed to tumor-derived peptides (a) or with gp96 complexed to peptides, peptides alone or PBS (b). Tumor growth was monitored. * is p<0.01 and ** is p<0.005 compared to α_2_M-irrelevant peptide group. Purifications of α_2_M were apparently homogenous as monitored by SDS-PAGE and immunoblotting with an α_2_M-specific antibody (inset).

To test the efficacy of α_2_M-peptide complexes in tumor immunotherapy, CMS5 were expanded in culture and used as a source of peptides [20]. The antigenically distinct MethA tumor was used as a source of irrelevant peptides. α_2_M was purified from serum of naïve BALB/c mice to apparent homogeneity as demonstrated by gel electrophoresis and immunoblotting (Fig. 1a, inset). α_2_M-peptide complexes were formed by very mild heat treatment as previously described [15], [20]. Cohorts of BALB/c mice were challenged CMS5 tumor cells and tumors were allowed to establish to 3×3 mm in diameter (day 3–4 post inoculation). Mice then received α_2_M-peptide complexes every two days for a total of 4 treatments (Fig. 1a). Control groups include mice treated with α_2_M complexed to irrelevant peptides, peptides alone or PBS. As a positive control, we used the immunogenic HSP, gp96, complexed in an identical manner to CMS5-derived peptides. Tumor growth was monitored. α_2_M (Fig. 1a) and gp96 (Fig. 1b) complexed to CMS5-derived peptides were able to elicit immune responses capable of significantly retarding the growth of CMS5 tumor cells. CMS5-derived peptides alone or α_2_M complexed to irrelevant peptides were unable to affect tumor growth demonstrating the requirement for the CD91/LRP-1-binding chaperone and the specificity provided by the peptides.

### Immunologically active α_2_M-tumor antigen complexes are pre-formed *in vivo*

**Figure 2:**
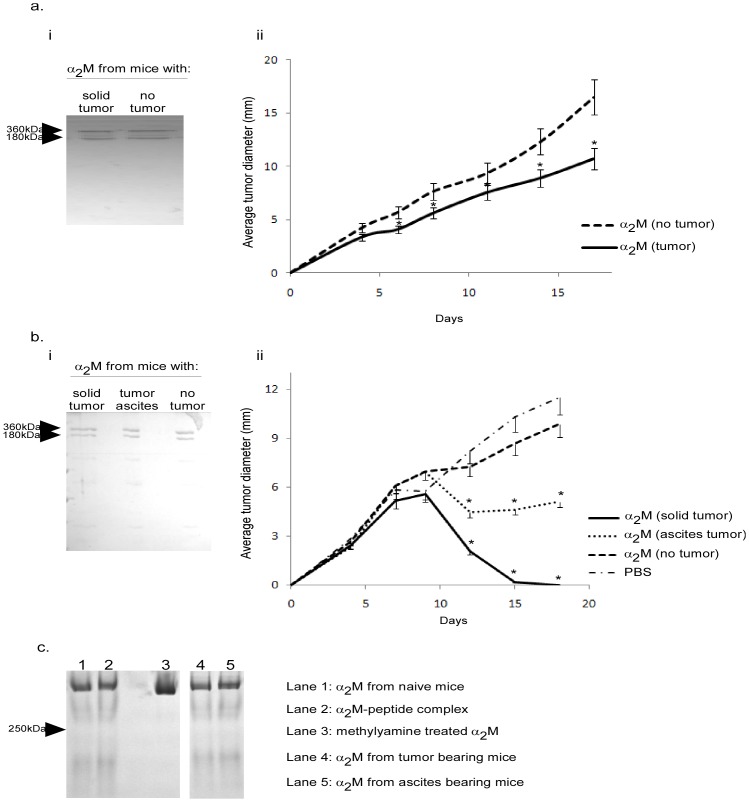
α_2_M from tumor-bearing mice is associated with antigen. a&b) α_2_M was purified from mice bearing solid tumors, tumors as ascites or from naïve mice. The purified α_2_M was monitored for purity by SDS-PAGE (a,i and b,i). a) Mice bearing established CMS5 tumors were treated with α_2_M that was derived from tumor bearing or naïve mice as indicated. Tumor growth was monitored. b) BALB/c mice were immunized with α_2_M that was derived from mice bearing solid tumors, tumors as ascites or from naïve mice (no tumor). One week later mice were challenged with MethA tumor cells. Tumor growth was monitored. p<0.05 between the two groups (a) or p<0.001 for each group compared to PBS (b). c) α_2_M purified from the indicated sources (lanes 1,4,5) were analyzed on 5% native polyacrylamide gels. α_2_M was additionally analyzed following complexing to peptides with mild thermal manipulation (lane 2). A “fast” form of α_2_M was made by treatment with methylamine (lane 3).

Previous immunological experiments with α_2_M were performed with α_2_M complexed to polypeptides *in vitro* [4], [6]. To examine if α_2_M-tumor antigen complexes form *in vivo*, CMS5 tumors were established in BALB/c mice and allowed to grow to 15 mm. Serum was harvested from these tumor-bearing mice and α_2_M was purified. Control α_2_M was purified from non-tumor-bearing mice. As established by electrophoresis, α_2_M from each group was apparently homogenous and identical with no other material co-eluting (Fig. 2a,i). Cohorts of BALB/c mice were then challenged with CMS5 and tumors were allowed to establish to 3×3 mm in size before being treated with α_2_M. Mice received α_2_M from tumor-bearing or non tumor-bearing mice every 2 days for a total of 4 treatments. Tumor growth was monitored (Fig. 2a,ii). α_2_M from tumor-bearing mice retarded the growth of tumors while α_2_M from non-tumor-bearing mice failed to do so. α_2_M from mice bearing irrelevant tumors also failed to affect tumor growth (data not shown). Since CMS5 tumors themselves do not express α_2_M, we predicted that tumors were shedding antigens into the microenvironment and beyond which could then be captured by α_2_M in the blood or extra-vascular space. To determine if the nature or site of the tumor affected the formation of the immunogenic complexes, we established MethA tumors as a solid intradermal tumor or as peritoneal ascites and tested the immunogenicity of α_2_M isolated from serum of mice bearing each type of tumor. α_2_M was purified from blood of mice bearing a dorsal ∼15 mm MethA tumor or bearing 4 day MethA ascites. Control α_2_M was purified from non-tumor-bearing mice (Fig. 2b,i). Cohorts of naïve BALB/c mice were immunized with α_2_M derived from the various donors twice one week apart. One week after the last immunization, mice were challenged with live MethA cells. Tumor growth was measured. Fig. 2b,ii shows that regardless of whether the Meth A was established as a solid tumor or as ascites the purified α_2_M was immunogenic and capable of eliciting tumor immunity as observed in the significant protection provided. However, it appears the α_2_M from solid MethA tumor-bearing mice was more efficient than α_2_M from ascites-bearing mice although when comparing these two groups one cannot easily compare the tumor volumes in the α_2_M-donor mice. To determine whether the conformation of α_2_M was altered and/or different in the tumor-bearing mice compared with the α_2_M from non-tumor bearing mice, α_2_M preparations were analyzed by native gel electrophoresis. There was no difference in electrophoretic mobility on native gels of α_2_M isolated from tumor bearing mice or non-tumor bearing mice (Fig. 2c). In addition, complexing of peptides to α_2_M *in vitro* did not alter the conformation of α_2_M. As a positive control, α_2_M was treated with methylamine which is known to convert α_2_M to a conformationally “fast” state as analyzed on a native gel [21]. Methylamine treated α_2_M is shown to migrate further down the native gel (Fig. 2c, lane 3).

### α_2_M elicits two signals required for T cell priming via APCs

**Figure 3:**
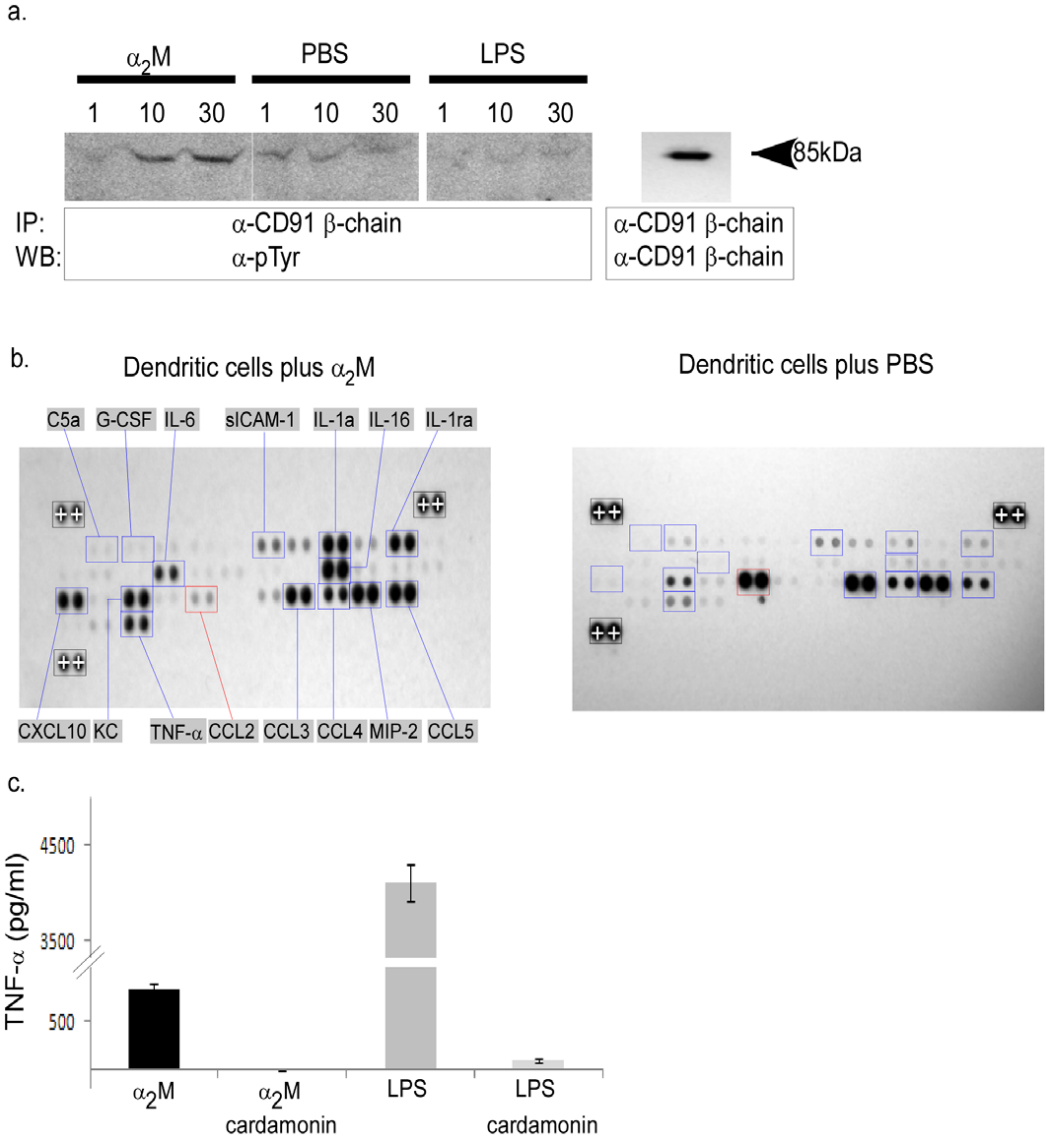
α_2_M provides signals necessary for T cell priming. a) RAW264.7 cells were pulsed with α_2_M, LPS or PBS. The β-chain of CD91/LRP-1 was immunoprecipitated and probed with α-pTyr antibodies. b) Splenic dendritic cells were pulsed with α_2_M (left panel) or PBS (right panel) for 20 h. Supernatants from either culture were incubated with cytokine profiler arrays as described in Methods and developed. Signals higher (blue boxes) or lower (red boxes) than background (PBS pulsed cells) are highlighted. Standardization signals on each blot (with+signs in Black boxes) are used to normalize signals to each other. c) Splenic dendritic cells were pulsed with α_2_M or LPS in the presence or absence of the NF-κB inhibitor cardamonin for 20 h. Supernatants were analyzed by ELISA for TNF-α. Error bars represent standard deviation of duplicates.

Priming of T cells requires the recognition of MHC-peptide complexes in the presence of co-stimulation. We tested if α_2_M could activate antigen presenting cells (APCs) to provide co-stimulation through its receptor CD91/LRP-1. The cell line RAW264.7, which has a high expression of CD91/LRP-1, was pulsed with α_2_M and phosphorylation of CD91/LRP-1 was monitored by immunoprecipitation and anti-phosphotyrosine immunoblotting. Cells were pulsed with LPS or PBS as controls. The β-chain of CD91/LRP-1, which is largely intracellular and harbors the 2 NPXY motifs [3], was probed. Fig. 3a shows that the β-chain of CD91/LRP-1 is phosphorylated in response to α_2_M. CD91/LRP-1 has only a baseline phosphorylation when pulsed with PBS or LPS. The increase in phosphorylation in response to α_2_M over PBS is significant when the intensities of the bands in the immunoblots are quantified (data not shown). We tested whether these signaling events led to the production of cytokines that promote T cell priming. Spleen-derived CD11c^+^ cells (representing splenic dendritic cells) were pulsed for 24 hours with α_2_M or PBS. Supernatants of the culture were harvested and probed with a cytokine array profiler. Results presented in Fig. 3b show a number of pro-inflammatory cytokines (IL-6, IL-1α, TNF-α) were significantly up-regulated when compared to cells pulsed with PBS. Impressively, there were several chemokines that were significantly changed including CXCL10, CCL2, CCL3, CCL4, CCL5, IL-16, and MIP-2. A number of other immune related molecules were also up-regulated including C5a, G-CSF, sICAM-1, IL-1ra, and KC. We tested whether one pro-inflammatory cytokine, TNF-α was produced in an NF-κB dependent manner. DCs were cultured with α_2_M in the presence or absence of cardamonin, a known NF-κB inhibitor. TNF-α production was effectively abrogated in the presence of cardamonin (Fig. 3c).

**Figure 4:**
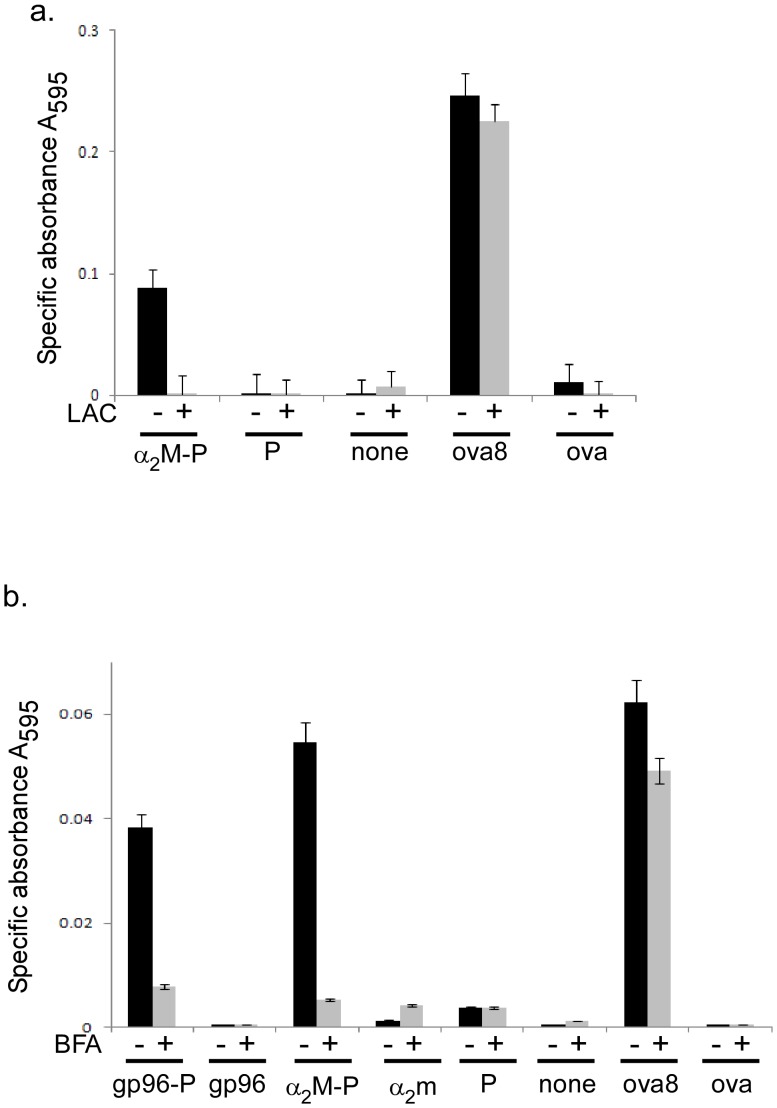
Cross-presentation of α_2_M-chaperoned peptides is dependent on proteasomes and vesicular trafficking. a) α_2_M complexed to the ova 19-mer peptide (P) was incubated with bone marrow-derived dendritic cells in the presence or absence of the proteasome inhibitor lactacystin. Controls include cells incubated with peptide alone, ova8 or ovalbumin protein (ova) or PBS (none). B3Z activation was monitored at A_595_ after addition of substrate. b) α_2_M or gp96 complexed to the ova 19-mer peptide (P) were incubated with bone marrow-derived dendritic cells in the presence or absence of BFA. Controls include cells incubated with ova 19-mer peptide alone (P), ova8 or ovalbumin protein (ova) or PBS (none). B3Z activation was monitored at A_595_ after addition of substrate. Error bars in (a,b) represent standard deviation of duplicates.

MHC I presents peptides 8–10 amino acids in length to T cells, and the proteasome is a major protease involved in trimming peptides to size. α_2_M has been shown to channel its chaperoned peptides for cross-presentation on MHC I [4]. The cross-presentation assays consist of bone marrow derived dendritic cells (BMDCs) cultured with α_2_M complexed to ova19-mer peptide. The ova19-mer peptide was used because this peptide requires trimming prior to presentation of SIINFEKL to B3Z T cell hybridoma. As shown in Fig. 4a, the ova19-mer peptide is capable of activating B3Z when chaperoned by α_2_M. We performed cross-presentation assays in the presence or absence of the proteasome inhibitor lactacystin to determine the requirement for the proteasome in α_2_M-mediated cross-presentation. The response was dependent on functional proteasomes because the signal was abrogated in the presence of lactacystin (Fig. 4a). The ova19-mer peptide by itself did not activate B3Z due to its length and inefficiency of uptake [22]. The ova8-mer, which does not require processing, is presented to B3Z in a proteasome independent manner as expected. We used ova protein as an additional control at equimolar concentrations. The protein was poorly taken up and processed due to the lack of a specific receptor. These results are identical to those obtained when peptides are chaperoned by gp96, an independent ligand for CD91/LRP-1 [2].

Peptides that enter the classical pathway of antigen processing and presentation by MHC I are further trimmed in the ER and packaged into vesicles for shuttling to the plasma membrane. Vesicular traffic is inhibited by brefeldin A (BFA). We performed the cross-presentation assays in the presence or absence of BFA. The cross-presentation of ova19-mer peptide chaperoned by α_2_M was dependent on vesicular traffic because BFA abrogated the robust signal observed in its absence (Fig. 4b). The same peptide when chaperoned by gp96 was also dependent on vesicular traffic. As in the previous figure, no signal was detected when ova protein, α_2_M or peptide were incubated separately with BMDCs.

## Discussion

We examined the effectiveness of α_2_M in cancer immunotherapy and observed that α_2_M-peptide complexes prime immune responses that retard the growth of established tumors in mice. Due to the difficulty in identifying tumor antigens and the corresponding antigenic peptides, we used the total repertoire of peptides that are generated in the tumor cells. In addition, we tested and showed that the association of tumor-derived antigenic peptides with α_2_M occurs naturally in mice as these complexes can be purified intact without manipulation *in vitro*. Finally we analyzed the ability of α_2_M to provide signals necessary for T cell priming and observed that APCs pulsed with α_2_M secrete pro-inflammatory cytokines, of which at least one, TNF-α, was in an NF-κB dependent manner. α_2_M-chaperoned peptides were cross-presented in a proteasome-dependent manner and cross-presentation was also dependent on vesicular traffic.

Our work on HSPs and α_2_M overlap with respect to their common receptor CD91/LRP-1 [1]–[3]. We have studied the immunobiology of HSPs and shown that these proteins elicit powerful immune responses that prevent or treat cancer. HSPs are currently being used in Phase III clinical trials in patients with melanoma and renal cell carcinoma [23], [24]. Like the HSPs, the unique use of CD91/LRP-1 for endocytosis and cross-presentation confers the ability of α_2_M to elicit T cell immune responses to peptides they chaperone, as shown by numerous independent groups [4], [6], [25]. In addition we have recently shown that CD91/LRP-1 serves as a signaling receptor for HSPs [26] and now we show here that the same is true for α_2_M. While prophylactic responses against tumor challenges are usually observed following priming of T cell responses with numerous reagents, therapeutic responses are much more difficult to achieve and in most cases actually fail. For example, immunization with tumor cell lysate primes robust T cell responses [27] but is ineffective in immunotherapy of cancer. We have thus been encouraged by the results presented here showing the effectiveness of α_2_M in immunotherapy of tumor disease in mice.

Studies by Klein and colleagues several decades ago [17] were unable to transfer anti-tumor responses between mice with serum and found activity only in the cellular components of blood. Since then serum has been characterized as immunologically silent. We were thus surprised to find immunologically active components in serum- the α_2_M-antigen complex. In our studies it appears that the access of tumor antigens to the blood may not be routine. More likely, access would depend on tumor morbidity and mortality, size, type, encapsulation, microenvironment and other factors. The presence of elevated prostate serum antigen (PSA) in blood of most prostate cancer patients appears to be at least one example of robust access of tumor-derived material to the blood. Indeed studies have found that majority of the PSA present in blood is in a complex with α_2_M [28]. The identification of bonafide and protective tumor antigens (that is whole protein or derivative peptides) in blood and the pre-formation of α_2_M-tumor antigen complexes would generate an effective vaccine for treatment of various cancers.

We have attempted to identify peptide repertoires isolated from α_2_M by mass spectrometry. At present we do not have a complete answer because of technical limitations. Essentially, as expected, the peptides we isolate are of a wide range and very low frequency, representing a large number of self (normal) proteins. Tumor antigens associated with α_2_M are expected to be a tiny percentage of the total repertoire of peptides and will be randomly extended variants of the final MHC I presented peptide. Overall signal intensity of precursor ion measurement for known tumor antigens (ERK2 peptide for CMS5) has thus been low and current efforts are underway to compare the sequence information of a few hits with synthetic analogs to increase confidence. However, we are able to measure specific immunological responses as reported here due to the inherent amplification steps during priming of T cell responses. We do not rule out the possibility that the tumor antigen bound by α_2_M may be a protein as α_2_M is known to chaperone a large family of proteins.

The long standing question then is whether α_2_M-antigen complexes self immunize in the host? The answer may be in three parts; first, α_2_M-tumor antigen complexes may indeed immunize the host, however tumor growth may progress because it becomes immuno-insensitive through a variety of mechanisms including loss of antigen and/or MHC or gain of suppressor functions. This is supported by the routine detection of anti-tumor T cells in cancer patients with progressive disease [29]. α_2_M-tumor antigen complexes may thus play a role in immuno-editing of the tumor phenotype [30]. Second, components in the serum may act antagonistically through blocking CD91/LRP-1 binding, production of T cell suppressor cytokines etc. Studies to fractionate and purify various serum components will reveal factors that are separately or synergistically immunogenic, or inhibitory, suppressive or counterproductive in the sum total. In our experiments, intradermal immunization with α_2_M-antigen complexes targets lymph nodes not exposed to potential antagonistic material in serum. Third, purification of α_2_M from serum leads to its activation. Indeed numerous investigators have proposed the idea that activation of α_2_M through proteolytic cleavage (during blood clotting) leads to a more robust interaction with CD91/LRP-1 [31]. We make a cautionary note to distinguish (proteinase) activation of α_2_M *in vivo* from methylamine treatment of α_2_M *in vitro*. By treating α_2_M with methylamine, two conformationally distinct states of α_2_M can be identified by native gel electrophoresis [21]. It has been suggested that the electrophoretically “fast” form of α_2_M binds to molecules given that, upon administration to animals, the “fast” form of α_2_M is rapidly cleared by the liver [32]. However, α_2_M has been known to bind cytokines, hormones and growth factors in a manner that is clearly independent of such “slow” and “fast” conformational conversion mechanisms [33]–[36]. Thus the relevance of these two forms of α_2_M *in vivo* has been questioned [31] and in our study here we do not observe any conformational changes in α_2_M isolated from tumor bearing mice compared to α_2_M from non-tumor bearing mice. The exact mechanism by which α_2_M, isolated from blood of tumor bearing mice, elicits tumor-specific immunity is under investigation.

The use of various endogenous molecules for elicitation of immune responses was pioneered by us and confirmed by many other groups. This has led to the use of these molecules (HSPs) in clinical trial for patients with cancer and infectious disease which are far advanced. [12], [13], [23], [24]. Our studies identify a new approach for vaccine design where the immunogenic α_2_M complexes can be purified intact from patients and used for vaccine purposes with the added benefit of the easy availability of that material.
